# Expression of TRPM8 in the distal cerebrospinal fluid-contacting neurons in the brain mesencephalon of rats

**DOI:** 10.1186/1743-8454-6-3

**Published:** 2009-03-17

**Authors:** Jing Du, Xinwei Yang, Licai Zhang, Yin-ming Zeng

**Affiliations:** 1 Jiangsu Province Key Laboratory of Anesthesiology, Xuzhou Medical College, Xuzhou 221002, PR China; 2 Department of Cardiothoracic Surgery, Changhai Hospital, Second Military Medical University, Shanghai 200433, PR China

## Abstract

**Background:**

It has been shown that distal cerebrospinal fluid-contacting neurons (dCSF-CNs) exist near the ventral midline of the midbrain aqueduct and also in the grey matter of the inferior third ventricle and the fourth ventricle floor in the superior segment of the pons. The dCSF-CNs communicate between the cerebrospinal fluid (CSF) and the brain parenchyma and may participate in the transduction and regulation of pain signals. The cold sensation receptor channel, TRPM8 is involved in analgesia for neuropathic pain, but whether the TRPM8 receptor exists on dCSF-CNs remains unknown. However, there is preliminary evidence that TRPM8 is expressed in dCSF-CNs and may participate in the transmission and regulation of sensory information between brain parenchyma and cerebrospinal fluid (CSF) in rats.

**Methods:**

Retrograde tracing of the cholera toxin subunit B labeled with horseradish peroxidase (CB-HRP) injected into the lateral ventricle was used to identify dCSF-CNs. A double-labeled immunofluorescent technique and laser scanning confocal microscopy were used to identify the expression of TRPM8 in dCSF-CNs. Software Image-Pro Plus was used to count the number of neurons in three sections where CB-HRP positive neurons were located in the mesencephalon of six rats.

**Results:**

The cell bodies of CB-HRP-positive dCSF-CNs were found in the brain parenchyma near the midline of the ventral Aq, also in the grey of the 3V, and the 4V floor in the superior segment of the pons. In the mesencephalon their processes extended into the CSF. TRPM8 labeled neurons were also found in the same area as were CB-HRP/TRPM8 double-labeled neurons. CB-HRP/TRPM8 double-labeled neurons were found in 42.9 ± 2.3% of neurons labeled by TRPM8, and all CB-HRP-labeled neurons were also labeled with TPRM8.

**Conclusion:**

This study has demonstrated that the cold sensation receptor channel, TRPM8, is localised within the dCSF-CNs of the mesencephalon. TRPM8 acts as receptor of dCSF-CNs for sensation transmission and pain regulation.

## Background

The transient receptor potential (TRP) channel is a transmembrane protein which is a thermo-sensitive channel that is expressed on sensory neurons and skin epithelial cells in vertebrate and non-vertebrate animals [1,2]. A subtype of TRP, TRPM8 which was originally cloned as a prostate-specific protein, has been widely known as a cold- and menthol-activated channel implicated in thermosensation. Recent studies have revealed that TRPM8 is not only necessary for cold sensation [3,4], but also mediates the process of analgesia for neuropathic pain activated by cold [5]. The results indicate that TRPM8 is related to cold-induced pain relief and may have some potential for pharmacological applications.

There are three types of CSF-CNs: intraependymal neurons which project into the ventricle lumen and the central canal of the spinal cord, supraependymal cells which are subjacent to the ependyma, and distal CSF-CNs [6,7]. Many studies have been made on the distribution of CSF-CNs in the parenchyma of the brain with horseradish peroxidase (HRP) and autoradiography. However, a significant amount of data has shown that both HRP and radiolabeled substances can pass freely through the ependymal lining of the ventricles into the parenchyma of the brain [7]. Because of this property, it has been necessary to develop an alternative neuronal marker that does not pass through the ependymal layer and will only label neurons with processes in the CSF. Zhang *et al *found that cholera toxin subunit B labeled with horseradish peroxidase (CB-HRP) was a dependable tracer for CSF-CNs [7]. After injecting CB-HRP into lateral ventricle (LV), it was found that there was a distinct outline labeled with CB-HRP on the ependymal surfaces of the ventricular system from LV, the intraventricular foramen, the third ventricle (3V), the midbrain aqueduct (Aq), fourth ventricle (4V) to the central canal (CC) and on the pial surfaces of the brain and spinal cord. The results indicated that CB-HRP does not pass through the spaces of the ependyma and diffuse into the parenchyma. Thus, any labeled structures found in the parenchyma of the brain must be CSF contacting structures. It was concluded that using CB-HRP as a tracer is a reliable method to label CSF-CNs [7,8].

Zhang *et al *also studied the distribution and the signaling directions of cerebrospinal fluid contacting neurons (CSF-CNs) in the parenchyma with CB-HRP tracing, combined with transmission electron microscopy [7]. The results were as follows: (1) CSF-contacting tanycytes were found not only in the wall of the 3V, but also in the walls of the LV, the 4V and the CC of the spinal cord. (2) Some CSF-contacting glial cells were observed in the lateral septal nucleus (LS). (3) Distal CSF-CNs in the parenchyma were found in LS, the anterodorsal thalamic nucleus (AD), the supramammillary nucleus (SuM), the dorsal raphe nucleus (DR), the floor of 4V and the lateral superior olive (LSO), but they were predominantly found in the mesencephalon region ventral to the aqueduct. (4) Axon terminals labeled by CB-HRP were found in the cavity of the brain ventricle. (5) The distal CSF-CNs whose properties are still under investigation, have a peculiar position in that their bodies are in the brain parenchyma distal to the ventricle system and their processes extend into the CSF. This suggests that they transmit signals between brain parenchyma and CSF. It is not known whether the signaling directions of the neuron are from CSF to the parenchyma, the parenchyma to CSF or both. Our previous experiment found that there were different synapses between CSF-CNs in mesencephalon region ventral to the aqueduct [7]. There were not only axo (-)-dendritic (+) synapses, but also dendro (-)-dendritic (+) synapses. However, their common characteristics were that the presynaptic elements were formed by non-CSF contacting neurons in the brain parenchyma, and the postsynaptic elements formed by the neurons in DR contacting the CSF. According to the general rule of chemical synapses, impulses are conducted from the presynaptic membrane to the postsynaptic membrane. Hence it was suggested that the signaling direction of the CSF-CNs in mesencephalon region ventral to the aqueduct occurs only from the brain parenchyma to the ventricular CSF [7]. Our previous studies indicated that dCSF-CNs may participate in transduction and regulation of pain signals [7,8]. TRPM8 function is related to the process of analgesia for neuropathic pain [5]; however, whether the specific pain signal receptor TRPM8 exists in dCSF-CNs remains unknown.

This paper, presents the first description and preliminary results of TRPM8 expressed on dCSF-CNs.

To determine whether the distal cerebrospinal fluid-contacting neurons possess the membrane receptor, TRPM8, we combined the cholera toxin B horseradish peroxidase (CB-HRP) retrograde tracing with TRPM8 immunofluorescence double-labeling technique to investigate the function of the dCSF-CNs.

## Methods

### Animal care and treatment

All experiments were conducted in accordance with the guidelines of the International Association for the Study of Pain (IASP) and approved by the Committee for the Ethical Use of Laboratory Animals, Xuzhou Medical College, license number: SYXK (Jiangsu) 2002–0038. Six male Sprague-Dawley rats (250 ± 50 g) were obtained from the experimental animal center, Xuzhou Medical College. The SPF grade rats were maintained in climate and light-controlled (23 ± 1°C, 12/12 h dark/light cycle with light on at 08:00 h) for at least one week prior to the experiments.

### CB-HRP injection

Rats were anesthetized with sodium pentobarbital (40 mg/kg, i.p.) and the head fixed in a stereotaxic instrument (Narishige Scientific Instruments, Tokyo Japan). A 3-μl volume of 30% CB-HRP (Sigma) was injected into one LV according to stereotaxic coordinates (Bregma: -1.2 ± 0.4 mm, Depth: 3.2 ± 0.4 mm, Right of median sagittal plane: 1.4 ± 0.2 mm).

### Tissue processing

Forty-eight h following tracer injection, rats were deeply anesthetized again with intraperitoneal pentobarbital sodium (50 mg/kg) and transcardially perfused with 150 ml of phosphate buffered saline (0.01 M PBS, pH 7.4), followed without interruption by 4% paraformaldehyde in 0.2 M phosphate buffer (300 ml, pH 7.4). The brainstem was removed immediately and post-fixed for 4–6 h at 4°C, then cryoprotected by immersion for 24–48 h in sucrose gradients (5%, 10%, 15%, 20%, and 30%) with 0.01 mol/L PBS at 4°C. The brainstem embedded with OCT, at -20°C and sectioned on a cryostat (Leica CM1900, Germany) at 25 μm in the transverse plane.

### Immunofluorescence procedures and confocal microscopy technique

The frozen sections were collected in PBS. Following 3 washes in PBS, sections were incubated in PBS with 0.3% triton X-100 (PBST) for 48–72 h at 4°C with an goat anti-cholera toxin B-subunit in (1:200, 227040, Calbiochem, San Diego, USA) and rabbit Anti-TRPM8 (ab3243, Abcam, Cambridge, UK). After rinsing in PBS, sections were incubated and in donkey anti-goat IgG-conjugated to tetraethyl rhodamine isothiocyanate (sc-2094 Santa Cruz Biotechnology), and in donkey anti-rabbit IgG conjugated to fluorescein isothiocyanate (sc-2090, Santa Cruz Biotechnology, Santa Cruz, USA), in the dark for 1 h at room temperature. Finally, sections were rinsed, mounted, and coverslipped with glycerol containing 2.5% of anti-fading agent DABCO (1, 4-di-aza-bi-cyclo-2, 2, 2-octane, Sigma, USA) and stored at -20°C in the dark. Tissue sections were examined using laser scanning confocal microscopy (TCS SP2, Leica, Wetzlar, Germany) to identify dCSF-CNs labeled with TRITC and TRPM8 labeled with FITC.

### Image analysis

Software Image-Pro Plus Version 6.0 (MediaCybernetics, Bethesda, USA) was used to count the number of neurons. Three sections with centralized CB-HRP positive neurons were chosen from the same aspect of brain parenchyma in each rat. Under 100× magnification sections 846.8 μm × 655.3 μm in area were selected to count the number of CB-HRP, TRPM8 and double-labeled CB-HRP/TRPM8-positive neurons. Data are expressed as mean ± SD.

## Results

### Location of labeled neurons

**Figure 1:**
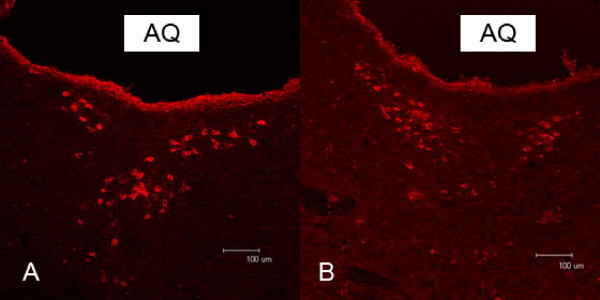
**Immunofluorescence of distal CSF-contacting neurons in rat mesencephalon**. Neurons labeled with tetraethyl rhodamine isothyocyanate (TRITC, red) after intraventricular administration of CB-HRP, were found in the mesencephalon ventral to the aqueduct. The number of labeled neurons varied in different sections (A, B). AQ: midbrain aqueduct. Scale bar: 100 μm.

The positive cells with labeling from the ventricular CB-HRP were observed as described by Zhang et al. [7,8]. These dCSF-CNs were found near the midline of the ventral Aq (Figure 1A and Figure 1B), also in the grey of the 3V, and the 4V floor in the superior segment of the pons.

### Image analysis and morphology

**Figure 2:**
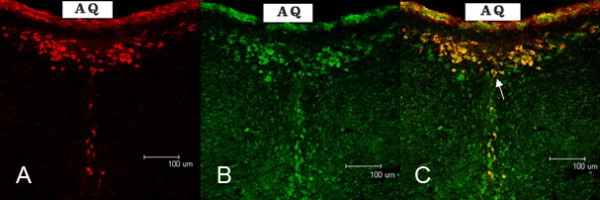
**Dual labelling of neurons with CB-HRP/TRPM8 fluorescent immunohistochemistry in rats**. A: CB-HRP positive neurons (red). The cell bodies of dCSF-CNs were in the brain parenchyma and the processes were extended into CSF. B: the same section showing TRPM8 positive neurons (green). C: same section showing CB-HRP/TRPM8 double-labeled neurons (arrow, yellow). All CB-HRP/TRPM8 double-labeled neurons were coincident with the neurons labeled by CB-HRP. Scale bar: 100 μm.

Positive labeling of the CB-HRP-traced neurons in the ventral aqueduct region was mainly located in cytoplasm, and the majority were multipolar and round or oval in shape. The cell bodies of these dCSF-CNs were in the brain parenchyma and the processes extended into CSF. A tissue section, CB-HRP immunoreactivity in red TRITC is illustrated in Figure 2A. TRPM8-positive neurons (FITC, green) were more numerous (Figure 2B). CB-HRP/TRPM8 double-labeled neurons were found in only a proportion of neurons labeled for TRPM8. On the other hand, all neurons labeled for CB-HRP were also CB-HRP/TRPM8-double-labeled neurons (Figure 2C).

### Neuron counting

Neurons were counted on three sections from each rat and the results are reported in Table 1. The mean number of double-labeled neurons in an area of 846.8 μm × 655.3 μm from the mesencephalon of six rats was 380 +/- 22. The mean number of TRPM8-labeled neurons for the same area was 884 +/- 19 or 42.9% of the double-labeled neurons.

**Table 1:** The number and means of positive neurons in the mesencephalon of six rats with double-labeling for CSF-injected CB-HRP and for TPRM8

**Rat number**	**CB-HRP and TRPM8 double-labeled (yellow)**	**TRPM8 (green)**	**CB-HRP/TRPM8(%)**
R1	405	909	44.6

R2	366	892	41.0

R3	389	861	45.2

R4	344	876	39.3

R5	395	898	44.0

R6	378	868	43.5

Mean ± SD	380 ± 22	884 ± 19	42.9 ± 2.3

All the brain tissue sections counted had CB-HRP/TRPM8 double-labeled neurons.

## Discussion

TRPM8 is a transient receptor potential cation channel which, after activation, is permeable to Ca2+ influx [9]. A previous study has suggested that TRPM8 not only conducts cold sensation but also has an important role in cold-induced pain relief [5]. TRPM8 was originally thought to be expressed almost exclusively in the prostate and in a number of non-prostatic primary tumours of breast, colon, lung and skin [10]. However, later studies detected TRPM8 mRNA or protein (or both) in a subset of sensory neurons from the DRG (dorsal root ganglion), trigeminal ganglia [11-13], nodose ganglion cells innervating the upper gut [14], gastric fundus [15], vascular smooth muscle [16], liver [17], in bladder urothelium, and different tissues of the male genital tract [18]. Recent studies have revealed that TRPM8 is not only necessary for cold sensation [3,4], but also mediates the process of analgesia for neuropathic pain after being activated by cold [5].

The dCSF-CNs were found near the midline of the ventral midbrain aqueduct (Aq) and also found in the grey of the third ventricle (3V) in the inferior and the fourth ventricle (4V) floor in the superior segment of the pons where neurons for pain regulation are concentrated [7]. Without special tracing method, the dCSF-CNs were hard to identify from the non-CSF-CNs in parenchyma of the brain. Up to present, their structure and function have been little investigated.

Both clinical practice and animal experiments indicate that the chemical composition of the CSF can change in pathological or special physiological conditions [19-22]. The reasons, origin and receptors for these chemical changes remain unclear. Our tracing experiment with intraventricular CB-HRP has shown that the bodies of CSF-CN in DR were in the brain parenchyma and their processes extended into CSF in the ventricle system. Our previous electron microscope observation showed that there were both excitatory and inhibitory synapses between non-CSF-CNs and CSF-CN in DR and found that the axon terminals of CSF-CN labeled by CB-HRP extended directly into the cavity of 3V [7]. Hence we can presume that when the CSF contacting neurons are stimulated in the brain by receiving signals, their axon terminals extend into the cavity of brain ventricles possibly to release some chemical substances into CSF and change the CSF composition. We speculate that the dCSF-CNs are specifically involved with the process of the substance transport, signal delivery, and function modulation between the brain and cerebrospinal fluid. The connection between CSF-CNs and CSF is via non-synaptic signal transmission, with the possibility that substances can be absorbed from the CSF as well as substances secreted into the CSF. In addition, there is the possibility that CSF-CNs could sense the pressure of CSF as well as changes in composition of various neurotransmitters and cell factors. Furthermore, dCSF-CNs can transmit the information to other areas of the brain [2,3]. CSF-CNs are always located on the ventral surface of mesencephalon aqueduct and within the brain parenchyma of the floor of the 4th ventricle, where the area of somatic pain sensation modulation resides.

The dCSF-CNs connect the CSF and the brain parenchyma, and have a more important function than the subependymal and ependymal CSF-CNs. Our previous studies have demonstrated that the dCSF-CNs are closely linked to inflammatory and neuropathic pain, and morphine dependency and withdrawal [7,8]. However, no previous studies have focused on the relationship between dCSF-CNs and cold and pain sensation. Therefore, we speculate that the dCSF-CNs participate in pain modulation via the cold sensation receptor TRPM8. This study demonstrates that the cold sensation channel, TRPM8, is expressed in the distal CSF-contacting neurons of mesencephalon ventral to the cerebral aqueduct. The existence of TRPM8 immunoreactivity suggests that CSF-CNs may transmit thermal and pain sensation to non-CSF-CNs in the central nervous system neurons via TRPM8.

Using the HRP tracing method [23,24], investigators found that the neurons in the mesencephalon region ventral to the aqueduct received many axis-cylinder contacts from many regions of the brain. These regions include the locus coeruleus nucleus, the solitary tract nucleus, the raphe magnus nucleus, the substantia nigra, the thalamo-central medial nucleus, the parafascicular nucleus, the gigantocellular reticular nucleus, the preoptic area and the cortex. Many studies have indicated that the neurons in the mesencephalon region ventral to the aqueduct have many physiological functions including thermoregulation [25], sleep [26-28], cardiovascular functions [29], hormone-endocrine functions [30,31], multiple arousal systems [32], obesity [33], anxiety and depression [34,35], conditioned-fear and stress [36], sexual behaviors [37] and mood disorders [38] and are also important in pain modulation and anti-nociceptive function [39,40]. Zhang *et al. *[7] indicated that the mesencephalon region ventral to the aqueduct was a locus which had the largest number of distal CSF contacting neurons and the most concentrated distribution. It is suggested that the neurons in the mesencephalon region ventral to the aqueduct also play an important role in signaling between the brain and CSF. In 1992, Wang & Zhang first used CB-HRP in retrograde tracing to label dCSF-CNs and obtained satisfactory results [41].

CB-HRP is an ideal dCSF-CN tracer which is absorbed by neurons via their receptors [8]. The sensitivity is 10 to 100 times higher compared to HRP [41]. A small dose applied to the peripheral nerves is sufficient to show the axis, dendrite, and neurons around peripheral nerve and its degradation time is of long duration. However, labeling the CNS using CB-HRP is poor, thus it has been rarely used. Based on our prior experiment [7], we assumed that the dCSF-CNs are different from the general central nervous neurons and their function is similar to that of peripheral neurons. TRPM8 is a cation channel that is specifically located on the peripheral receptors and is expressed in the dCSF-CNs. Our results indicate that the dCSF-CNs may be similar in function to peripheral sensory neurons. In addition, these findings provide a novel method for exploring the function and nature of dCSF-CNs.

## Conclusion

Our data suggest that the cold sensation receptor channel, TRPM8, is expressed in the nucleus of dCSF-CNs. The dCSF-CNs may play the important roles in neuromodulation and neuroendocrine regulation between brain parenchyma and CSF.

## List of abbreviations

CB-HRP: cholera toxin B conjugated to horseradish peroxidase; CSF-CNs: Cerebrospinal fluid-contacting neurons; dCSF-CNs: distal CSF-CNs; TRP: Transient receptor potential.

## Competing interests

The authors declare that they have no competing interests.

## Authors' contributions

JD and XY contributed equally to this work. JD carried out the experiments, performed data acquisition and part drafted the manuscript. XY was involved with interpretation and manuscript revision. LZ conceived and designed the project, revised experiments and drafted the manuscript. YZ was involved with manuscript revision. All authors read and approved the final manuscript.
